# Supplementary figures and images for: Geographic Distribution of Common Vampire Bat Desmodus rotundus (Chiroptera: Phyllostomidae) Shelters: Implications for the Spread of Rabies Virus to Cattle in Southeastern Brazil
Source: Pathogens. 2022 Aug 19;11(8):942. doi: 10.3390/pathogens11080942 (PMC9412479; doi:10.3390/pathogens11080942)

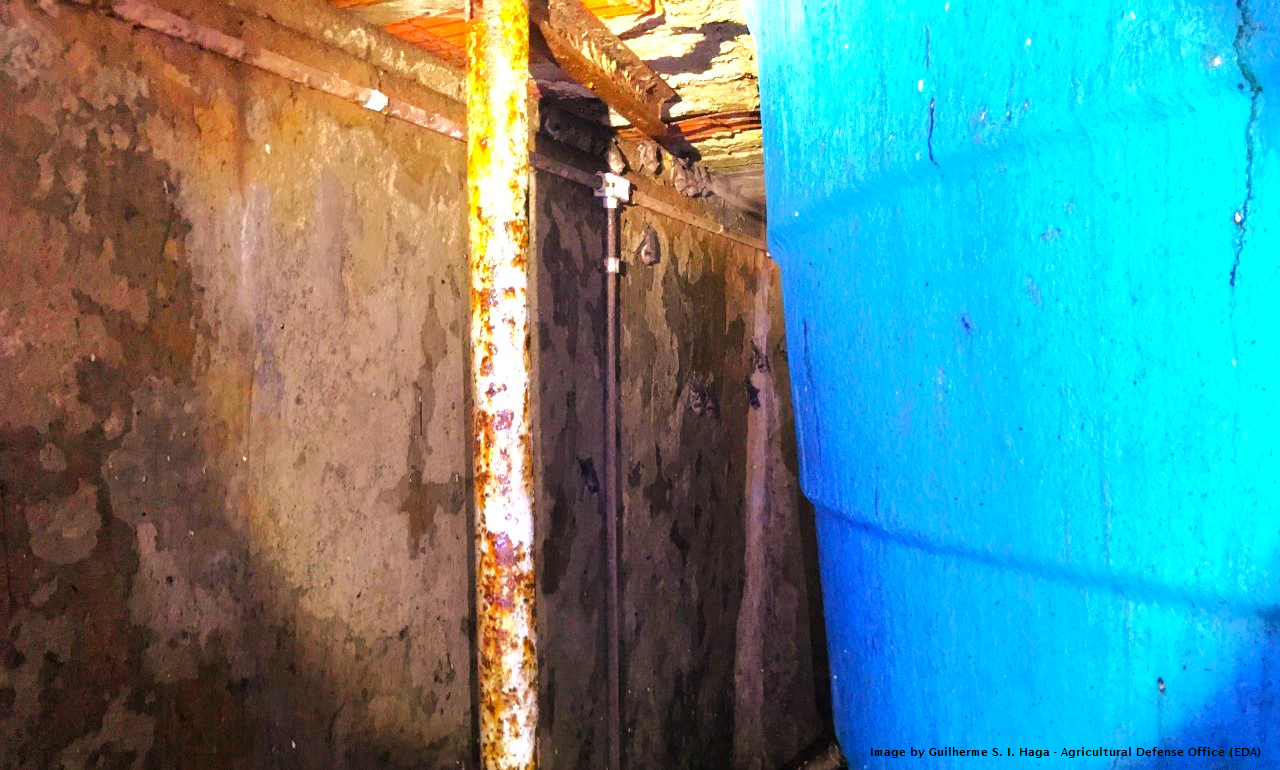

Supplement: Supplementary file 1 [file pathogens-11-00942-s001.zip › Figure S1 D. Rotundus water tank (abandoned house).png]

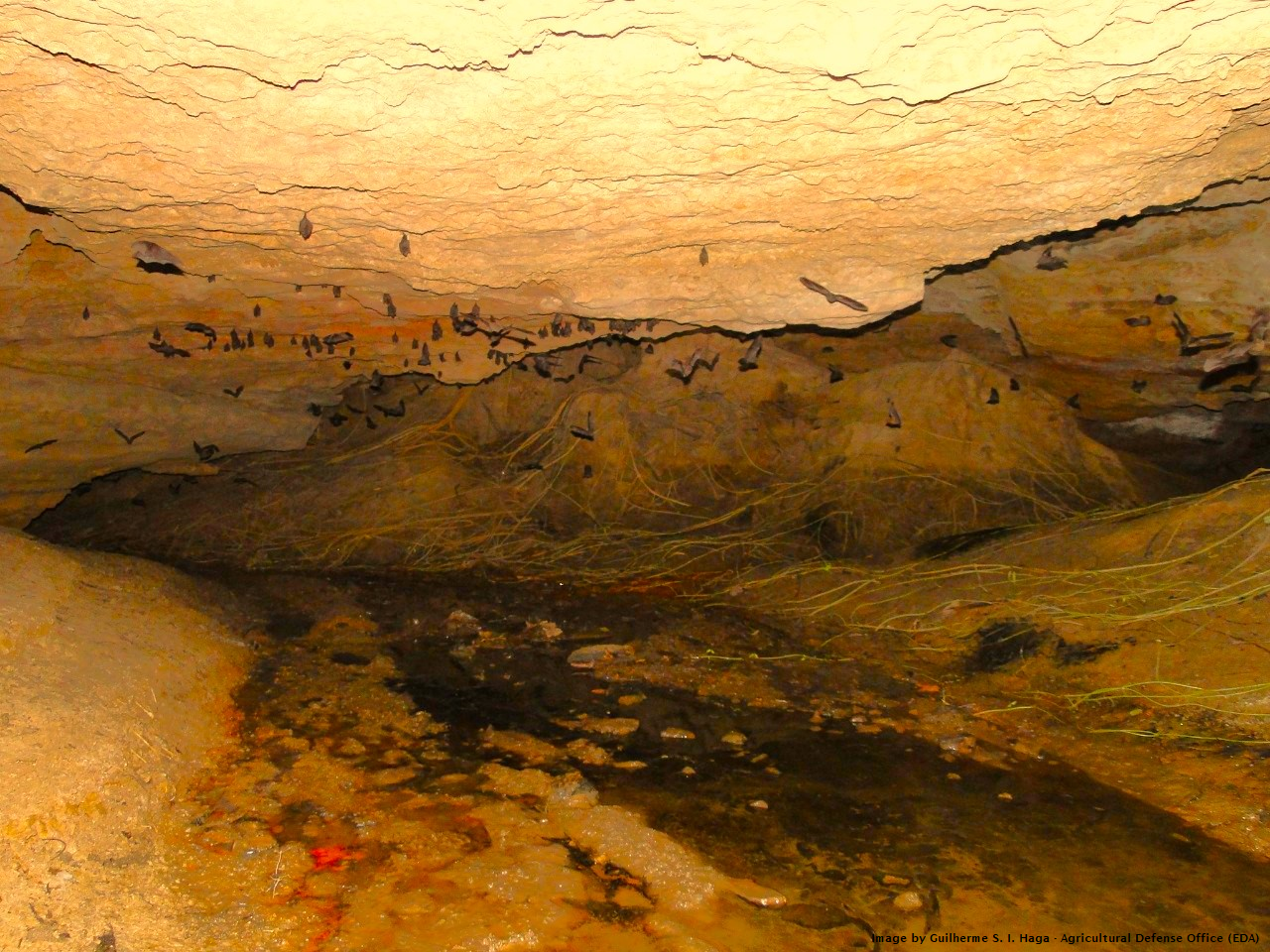

Supplement: Supplementary file 1 [file pathogens-11-00942-s001.zip › Figure S2 D. Rotundus grotto (cave) (1).png]

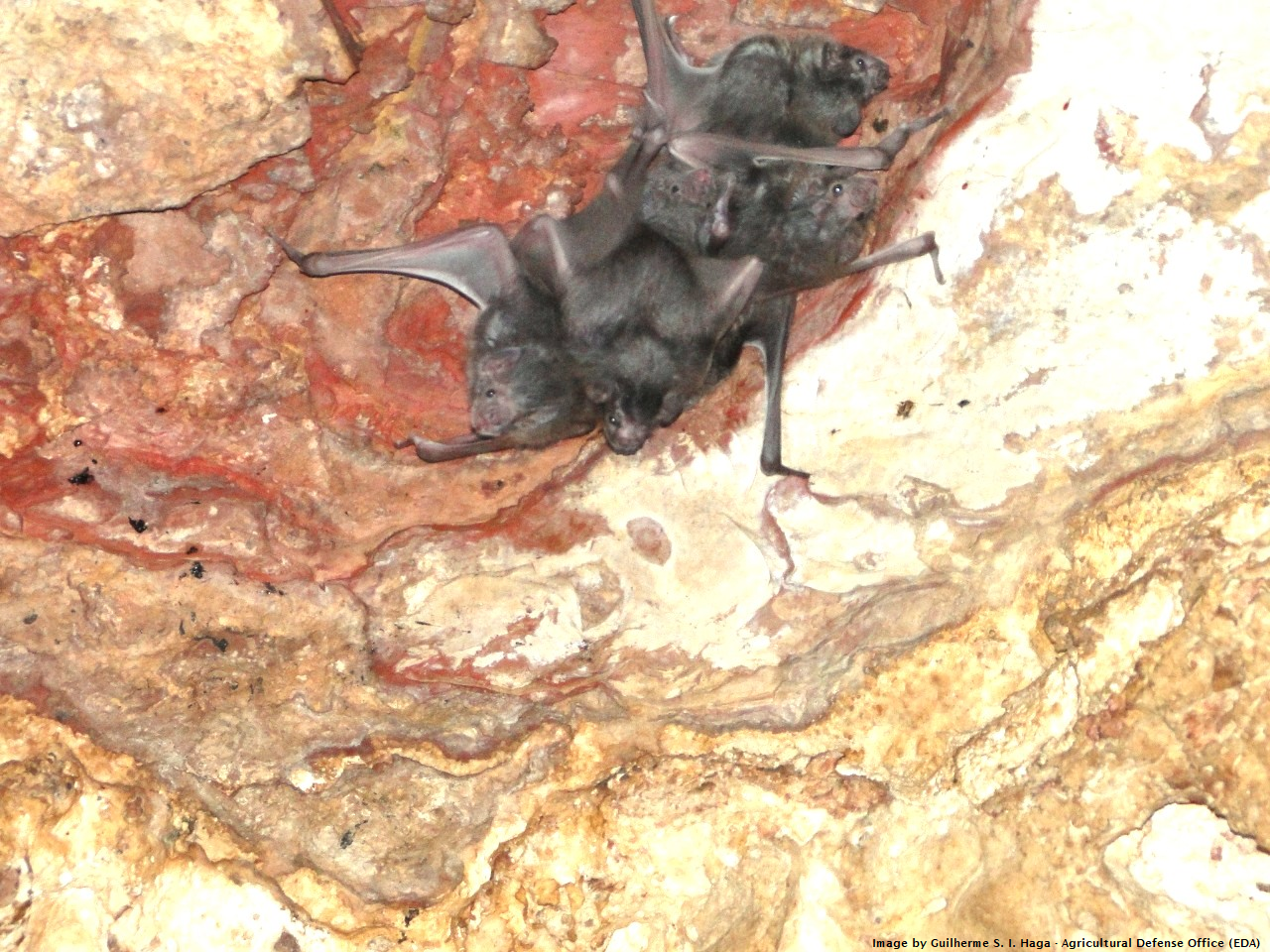

Supplement: Supplementary file 1 [file pathogens-11-00942-s001.zip › Figure S3 D. Rotundus grotto (cave) (2).png]
